# Wearing the face mask affects our social attention over space

**DOI:** 10.3389/fpsyg.2022.923558

**Published:** 2022-08-04

**Authors:** Caterina Villani, Stefania D’Ascenzo, Elisa Scerrati, Paola Ricciardelli, Roberto Nicoletti, Luisa Lugli

**Affiliations:** ^1^Department of Philosophy and Communication, University of Bologna, Bologna, Italy; ^2^Department of Biomedical, Metabolic and Neural Sciences, University of Modena and Reggio Emilia, Modena, Italy; ^3^Department of Psychology, University of Milano-Bicocca, Milan, Italy; ^4^Milan Centre for Neuroscience, University of Milano-Bicocca, Milan, Italy

**Keywords:** face mask, social cognition and interaction, gaze-cueing, Simon effect, COVID-19

## Abstract

Recent studies suggest that covering the face inhibits the recognition of identity and emotional expressions. However, it might also make the eyes more salient, since they are a reliable index to orient our social and spatial attention. This study investigates (1) whether the pervasive interaction with people with face masks fostered by the COVID-19 pandemic modulates the processing of spatial information essential to shift attention according to other’s eye-gaze direction (i.e., gaze-cueing effect: GCE), and (2) whether this potential modulation interacts with motor responses (i.e., Simon effect). Participants were presented with face cues orienting their gaze to a congruent or incongruent target letter location (gaze-cueing paradigm) while wearing a surgical mask (Mask), a patch (Control), or nothing (No-Mask). The task required to discriminate the identity of the lateralized target letters by pressing one of two lateralized response keys, in a corresponding or a non-corresponding position with respect to the target. Results showed that GCE was not modulated by the presence of the Mask, but it occurred in the No-Mask condition, confirming previous studies. Crucially, the GCE interacted with Simon effect in the Mask and Control conditions, though in different ways. While in the Mask condition the GCE emerged only when target and response positions corresponded (i.e., Simon-corresponding trials), in the Control condition it emerged only when they did not correspond (i.e., Simon-non-corresponding trials). These results indicate that people with face masks induce us to jointly orient our visual attention in the direction of the seen gaze (GCE) in those conditions resembling (or associated with) a general approaching behavior (Simon-corresponding trials). This is likely promoted by the fact that we tend to perceive wearing the mask as a personal safety measure and, thus, someone wearing the face mask is perceived as a trustworthy person. In contrast, people with a patch on their face can be perceived as more threatening, therefore inducing a GCE in those conditions associated with a general avoidance behavior (Simon-non-corresponding trials).

## Introduction

Humans are embedded in a social context and typically spend a significant amount of time interacting with other individuals and with objects in their environment. The spread of the COVID-19 pandemic has required large-scale habits change, ranging from self-isolation and social distancing to the pervasive use of the surgical face mask in everyday life. By heavily transforming the context surrounding us, COVID-19 has consequently transformed the way we interact with others. For instance, it has become relevant to assess whether the distance between us and the others is appropriate and, more importantly, whether other individuals near us are wearing the facemask or not.

While surgical masks have had a positive effect on preventing virus transmission (Liang et al., 2020), it seems that, by occulting a large portion of the human face, they interfere with the processing of key information that supports social interactions. Research conducted during the pandemic has highlighted that face-covering hampers face identification and perception (for a review, see Pavlova and Sokolov, 2022). Several studies revealed that face masks affect identity recognition, by altering face perception abilities (e.g., Freud et al., 2020; Noyes et al., 2021) and by reducing discrimination between familiar and unfamiliar faces (Carragher and Hancock, 2020). Others have shown that face masks diminish the ability to accurately classify emotions and facial expressions. For example, Carbon (2020) found that many emotional states, such as happy, sad, and angry were misinterpreted as neutral when the mask covered the lower face parts (see also Parada-Fernández et al., 2022; for theoretical discussion, see Nestor et al., 2020). Similarly, evidence from studies on scholar-aged children (Carbon and Serrano, 2021; see also Ruba and Pollak, 2020) revealed that masked faces impaired children’s emotional reading abilities to a different extent; with a strong effect on negative emotions, such as disgust, fear, and sadness, and a relative or null impairment in recognition of anger and neutral expressions. Marini et al. (2021) compared the ability to recognize attributes of faces when these were presented without the mask, with a standard surgical mask, and with a transparent surgical mask. They found that the transparent surgical mask, but not the standard mask, facilitated the recognition of emotional expressions and enhanced trustworthiness judgments; however, transparent masks impaired the re-identification of the same unmasked face, similar to the standard masks.

Interestingly, face masks impact not only identity and emotions recognition but also persons’ perception and social trait judgments. Olivera-La Rosa et al. (2020) asked participants to rate the perceived trustworthiness and sickness of, and desired social distance (i.e., social distance scale, Bogardus, 1933) from target faces wearing the surgical mask or not. They found that, compared to standard face target stimuli, faces wearing a surgical mask were perceived as more likely to be ill, but at the same time also as more trustworthy and more socially desirable for having closer interactions. The authors interpreted these results as a consequence of the internalization of social norms of wearing masks and keeping social distance imposed by the pandemic, which resulted in judging mask-wearers as more responsible and socially compliant, thereby promoting approach behaviors towards them. Similarly, Oldmeadow and Koch (2021) showed that masks increased the perceived trustworthiness and attractiveness of both Black or White faces, suggesting that the positive value of face masks is not influenced by racial profiling. In contrast, other studies have highlighted a negative bias in trustworthiness appraisals of masked faces. For example, Malik et al. (2021) showed videos of masked or unmasked actors offering economical advice to more than 2000 US citizens and found that only 5% trusted the advice given by masked strangers than when it was given by the unmasked strangers. In a rating study by Biermann et al. (2021), masked faces were evaluated as less trustworthy and less happy than unmasked faces; however, this effect was attenuated in participants who experienced high psychological distress and risk perception associated with the pandemic, and who showed high compliance with prevention measures to avoid infection. In a set of experiments, Twele et al. (2022) pointed out that facial masks might have a limited impact in forming first impressions of unfamiliar faces from across the lifespan. Specifically, they found that young adult faces with happy expressions were rated as more trustworthy than neutral faces, even when the same face had been previously seen with a mask; and that the presence of masks does not affect the adult’s perceived niceness of childs’ faces and the trustworthiness and competence of older adults face. The studies described above yielded contrasting results on whether and to what extent face masks affect interpersonal trust, likely due to the fact that they used different methodologies and stimuli. Nevertheless, the topic is of paramount interest and it might be relevant to investigate the impact of face masks on other social and cognitive processes.

Furthermore, it is worth noting that by covering the lowest part of the face, the face mask also increases the relevance of the eyes, a region that is known to play a particular role in human social interactions by providing a rich source of information to infer other’s intentions, emotional and mental states (e.g., Baron-Cohen et al., 2001; for recent reviews, see Grossmann, 2017; Capozzi and Ristic, 2018) and more generally to orient our own attention toward others (see Dalmaso et al., 2020).

The present work intends to explore whether wearing face masks that leave only the eyes region visible could impact social cognition, and more specifically two well-known attentional mechanisms, such as the gaze-cueing effect (GCE; for a review, see Frischen et al., 2007) and the Simon effect (e.g., Simon and Rudell, 1967; Simon, 1990; for a review, see Rubichi et al., 2006).

The GCE, a highly sensitive and reliable index of social attention, refers to the automatic tendency to shift attention towards the spatial location indicated by a task-irrelevant face with an averted gaze. In the standard gaze-cueing paradigm, participants are presented with a cue face on the screen that first looks straight ahead and then turns its gaze to the left or the right side. Shortly after this gaze shifting, a target letter either appears on a side congruent or incongruent to the gaze direction. Participants are asked to respond as quickly as possible to the identity of the target letter by pressing a button on the keyboard. People typically respond faster to targets appearing in the same location gazed at by the cue face (i.e., gaze-congruent trials) than to targets appearing in a location opposite to that gazed at by the cue face (i.e., gaze-incongruent trials), even though gaze direction is irrelevant to the task, thus indicating an automatic nature of social attention (Friesen and Kingstone, 1998; Driver et al., 1999; Langton and Bruce, 1999; Dalmaso et al., 2020).

The Simon effect (Simon, 1969, 1990) is characterized by a faster and more accurate performance when stimulus position and response position spatially correspond (i.e., corresponding condition) compared to when they do not correspond (i.e., non-corresponding condition), even though stimulus position is irrelevant to the task (e.g., Pellicano et al., 2009, 2019; Baroni et al., 2012; Lugli et al., 2013, 2016, 2017; Scerrati et al., 2017; D’Ascenzo et al., 2018, 2020). This difference in performance is thought to emerge because the stimulus location, although irrelevant, is being processed and leads to the automatic activation of the response that spatially corresponds with it. Therefore, in corresponding trials, the automatically activated response corresponds to the response required by task instructions, thus producing a more efficient performance. Conversely, in non-corresponding trials, the automatically activated response conflicts with the one indicated by task instructions, leading to slowed response times and increased errors (e.g., dual-route model, Kornblum et al., 1990; De Jong et al., 1994).

It is worth emphasizing that the GCE and the Simon effect have at least two main characteristics in common. The first is the need to extract a spatial code: for the GCE in order to direct the attention towards the gaze of the cue stimuli, and for the Simon effect in order to automatically process the position and the correspondence between stimulus and response. Second, both the effects can be modulated by different social factors. As for the GCE, the social factors previously investigated concern the observer, the cueing face or their specific relation. In particular, the face gender (e.g., Bayliss et al., 2005); age (e.g., Slessor et al., 2010, see also Ciardo et al., 2014); shared group-membership (e.g., Pavan et al., 2011; Dalmaso et al., 2014); competitive or cooperative behaviours (Ciardo et al., 2015); the perceived dominance and status (e.g., Jones et al., 2010; Dalmaso et al., 2012; Ciardo et al., 2021); trustworthiness (e.g., Süßenbach and Schönbrodt, 2014; Mattavelli et al., 2021); and emotional expressions (e.g., Bonifacci et al., 2008; Ricciardelli et al., 2012, 2016; for a recent review, see Dalmaso et al., 2020). Indeed, all these social factors have been shown to modulate the magnitude of the GCE. For example, stronger gaze-cueing effects were observed in response to faces described as belonging to trustworthy individuals compared to untrustworthy ones (Süßenbach and Schönbrodt, 2014) or when the gaze was perceived as more familiar (Deaner et al., 2007). However, Carraro et al. (2017) also showed that faces associated with negative/antisocial behaviors triggered stronger GCE than faces associated with positive/prosocial behaviors, and this effect was marked for participants who evaluated antisocial behaviors more negatively. Overall, it has been widely recognized that facial features convey crucial information for social interaction and provide cues to guide our behaviors towards others.

As for the Simon effect, several studies reported modulation of the effect according to the social relation between participants. In particular, Sebanz et al. (2003) were the first to show that the effect occurs even when the task is shared between two participants and different social factors are manipulated (e.g., Hommel et al., 2009; Iani et al., 2014, 2021; Lugli et al., 2015; Ciardo et al., 2016; Ruissen and de Bruijn, 2016). For example, some studies investigated the influence of interpersonal relationships: positive/bad mood or positive/negative relationship with the co-actor (i.e., participants with whom the task is shared), competitive/cooperative instructions to participants modulated the occurrence or the magnitude of the effect (e.g., Hommel et al., 2009; Kuhbandner et al., 2010; Iani et al., 2011; for a review Dolk et al., 2014).

Given the increasing amount of evidence showing how wearing facemasks profoundly affects our ability to regulate efficient social interactions, we aimed at exploring whether the presence of the surgical mask on a face impacts attention to the same extent as other social factors that previous works reported as successful in influencing the GCE and the Simon effect. The present work implemented a gaze-cueing paradigm where a horizontal left/right keypress response set has been used in order to investigate at the same time two effects (i.e., GCE and Simon). It is worth noting, in fact, that research implementing a gaze-cueing paradigm typically adopts a vertical up/down keypress response set to avoid any concurrent variance that might be produced by the relation between stimulus position and stimulus response (see, for example, Driver et al., 1999). In the few studies implementing a gaze-cueing paradigm where a horizontal left/right keypress response set has been used, the relation between stimulus position and stimulus response has not been taken into account and analyzed systematically (e.g., Dalmaso et al., 2012, 2014; Carraro et al., 2017).

More specifically, we conducted an online study in which the participants were shown a set of cue faces with averted gaze, looking either toward the left or toward the right. They were asked to perform a gaze-cueing task by pressing a lateralized response key associated with a specific target letter. The target could appear in a lateral position that could be either congruent or incongruent to the direction of gaze, which was irrelevant to the task. Each face was presented with a surgical facemask (i.e., Mask condition), without a mask (i.e., No-Mask condition), and with a patch that covered the same area occupied by the mask (i.e., control condition). The latter condition was introduced to allow us to exclude the possibility that a potential difference between the Mask and the No-Mask conditions was due to perceptual and spatial characteristics of having the mouth and part of the nose covered. In other words, in the control condition, the same face area, not visible in the Mask condition, was covered by a patch (with no intending protective meaning) to control for other spatial factors but leave the crucial eye region visible. Indeed, previous studies (e.g., Akiyama et al., 2008; Hayward and Ristic, 2015; Slessor et al., 2016) have shown that presenting just the eye region showing an averted gaze was enough to elicit the GCE. Therefore, our control condition as well as controlling for other perceptual factors allowed us to test whether the meaning of wearing a mask had a specific effect on gaze cueing.

Given the role of the mask in protecting ourselves and others from the spread of COVID-19, and building on the results of previous studies (e.g., Olivera-La Rosa et al., 2020), we considered the surgical mask as a positively valued object. For this reason, we hypothesized that participants could be more inclined to orient toward the direction of gaze of a person when this person is wearing a mask than when s/he is not wearing it, leading to a larger GCE in the Mask condition compared to the No-Mask condition and the Control condition. More specifically, we reasoned that observing someone who is wearing a facemask could lead us to orient our attention more in the direction of his/her gaze (thus to a greater GCE) than observing someone who is not wearing it as we may feel, for example, much more well-disposed towards those we do not perceive as a threat to our health. Alternatively, or as well, the facemask could lead to a greater GCE as the person wearing it could be perceived as more reliable than others thus deserving our trust.

As for the classic Simon effect, we hypothesized that the type of conditions (i.e., mask, no mask, control) should have a weaker effect, or no effect at all, since the classical Simon effect has not been reported to be affected by the participant’s attitude towards the stimulus, or by social perception, since it does not require to take into account a shared spatial representation of the task. By adopting a horizontal left/right keypress response set we were allowed to explore whether any spatial interplay (due to processing of a spatial code in both tasks) occurred between the GCE and the stimulus–response correspondence, and whether it varies across the manipulated conditions. More specifically, we aimed at examining whether the conflict that originates from the Simon effect has an influence on the GCE. Indeed, as far as we know, there are only a few previous studies that employed an orthogonal manipulation of stimulus position and gaze direction and found the two effects being independent (Zorzi et al., 2003; see also Ricciardelli et al., 2007). However, these previous studies did not employ the classic gaze-cueing paradigm and used schematic eyes rather than realistic gaze stimuli.

In summary, this study aims at assessing whether the presence of a surgical mask on the face modifies the gaze cueing effect, and whether and how this form of social attention could be modulated by motor conflict (like the ones posited by Simon tasks). Understanding the mechanisms underlying the interaction between social attention and motor behavior is a timely and crucial issue. Since the COVID-19 global pandemic hugely impacted humans’ social relations, it is important to understand whether the use of the face mask, useful to protect ourselves and prevent the spreading of the disease, has altered our perception and disposition towards the other.

## Materials and methods

### Participants

Data were collected from June 19, 2020, until December 8, 2020, between the first and second waves of the pandemic in Italy. We calculated the sample size required to achieve 80% power to detect a significant Congruency (i.e., GCE, congruent vs. incongruent) × Correspondence (i.e., Simon effect, corresponding vs. non-corresponding) × Condition (Mask, No-Mask, Control) interaction with the G*power 3.1 (Faul et al., 2007) software. With an effect size *f* = 0.15 (Cohen, 1988) and a correlation among repeated measures = 0.5, the power calculation yielded a recommended sample size of at least 42 participants.

A total of 40–60 undergraduate Italian students (40 females; 16 males; 7 left-handed; M age = 20.2 years; SD age = 2.5) from the University of Bologna participated as volunteers. All were naïve as to the purpose of the experiment. The study was conducted in accordance with the ethical standards laid down in the Declaration of Helsinki, and fulfilled the ethical standard procedure recommended by the Italian Association of Psychology (AIP). Written consent was obtained from all of them.

### Apparatus and stimuli

We used the online behavioral science platform Gorilla (www.gorilla.sc; Anwyl-Irvine et al., 2020; for a critical overview of the online platform see Scerrati et al., 2021) to create and host the experiment. In order to minimize possible distractions, the participants were invited to carry out the experiment in a quiet place and to avoid manipulating objects during the entire task. In addition, we asked participants to close other background apps/programs and all browser windows except for that of the experiment. The automated procedure ensured that participants were all using computers, since no other devices were allowed (e.g., tablets, smartphones), and automatically rejected participants who took longer than 2 h to complete the task.

Stimuli were grayscale photographs (198 × 283 pixels) depicting 4 Caucasian young adults (2 females and 2 males) bearing a neutral expression. All photographs, selected from the Karolinska Directed Emotional Faces set (KDEF; Lundqvist et al., 1998), had a direct gaze and the versions with the averted gaze were taken from Ricciardelli et al. (2012, 2016), who already manipulated this aspect in their study starting from the same (identity faces: AF21 AF31 AM10 AM17). Informed consent for publication of identified images is available at https://kdef.se/home/using%20and%20publishing%20kdef%20and%20akdef.html.

**Figure 1:**
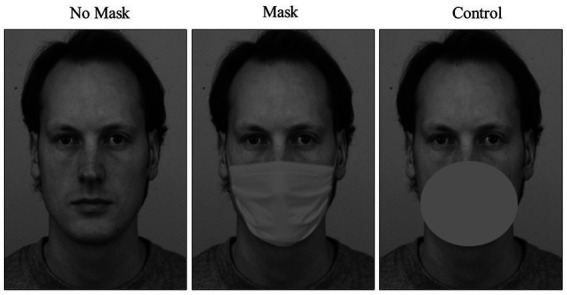
Example of the three conditions employed in the study: No Mask, Mask, and Control. Face stimuli were selected from the Karolinska Directed Emotional Faces database (KDEF; Lundqvist et al., 1998). The identity of the face depicted is AM10 NEU.

Adobe Photoshop software was used to create a grayscale mask (129 × 122 pixel) and a patch (106 × 93 pixel), that were superimposed in the lower part of each face stimulus, to create the stimuli conditions Mask and Control, respectively. The No-Mask condition refers to face stimuli with no element superimposed. Thus, the final set of face stimuli consisted of 24 pictures: 4 different individuals (2 females and 2 males) × 2 gaze direction (left and right) × 3 conditions (Mask, No-Mask, and Control). An example of the stimuli used in the experiment is displayed in Figure 1.

### Procedure

**Figure 2:**
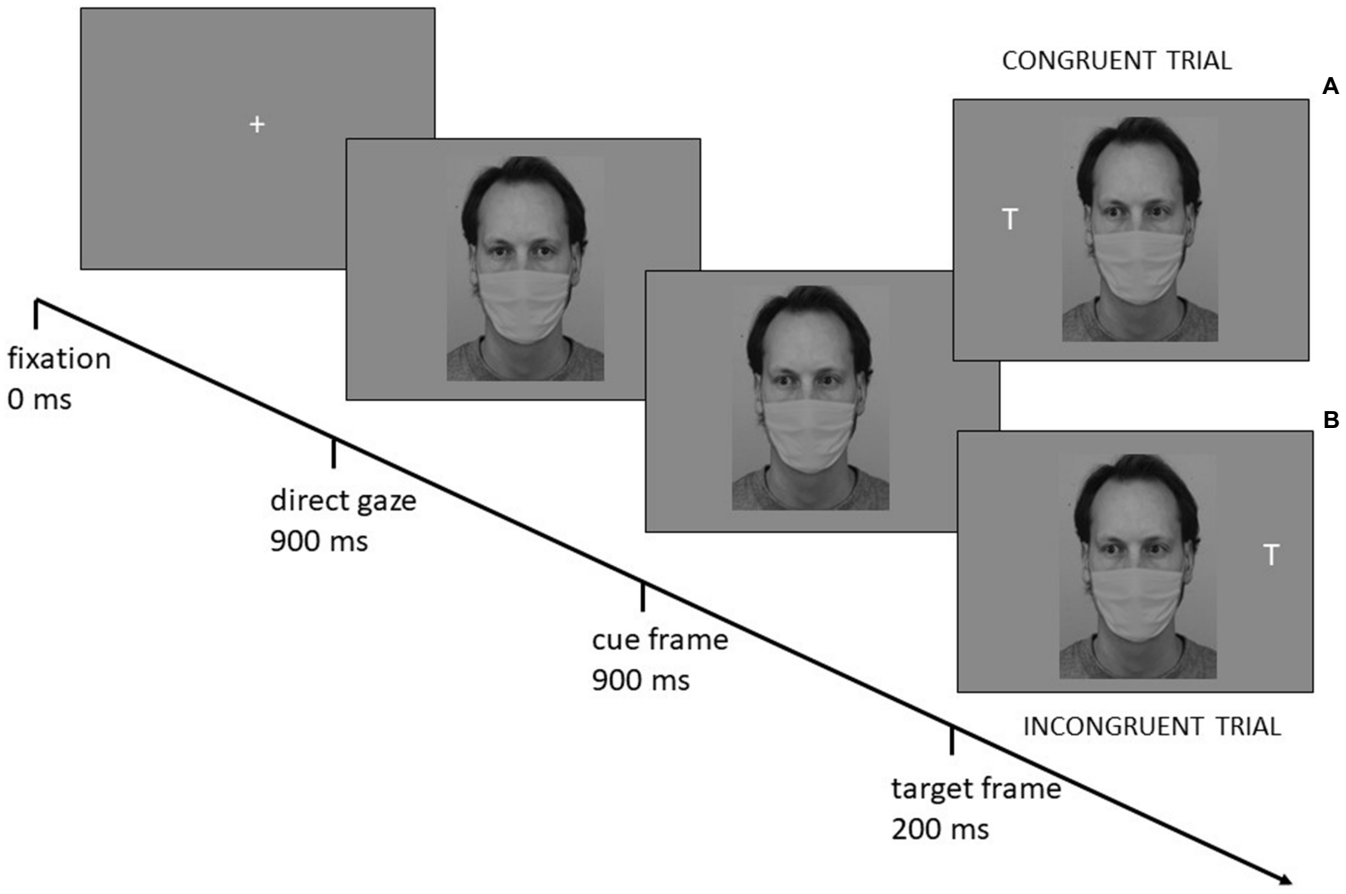
Illustration of the gaze-cueing procedure depicting examples of Mask condition stimuli (not drawn to scale) and sequence of events for a congruent trial (top) and an incongruent trial (bottom).

Each trial began with the presentation of a white fixation cross (23 × 23 pixel) in the center of a gray screen for 900 ms, followed by a central face with a direct gaze. After 900 ms, the same face appeared with an averted gaze (cue frame). After 200 ms, a white target letter (L or T, about 18 × 30 pixel, target frame) appeared to the left or right of the cue face, with equal probability. The target location could be spatially congruent or incongruent with the gaze direction and spatially corresponding or non-corresponding with the response location. The target frame remained visible until a response was provided (Figure 2; see Dalmaso et al., 2012; Ciardo et al., 2015 for a similar gaze-cueing procedure). The gaze direction was uninformative relative to the target location. Participants were instructed to maintain the fixation at the center of the screen and to respond according to the letter identity by using their right and left index fingers. Half of the participants were instructed to press a left key if the target was an “L” and a right key if the target was a “T” (respectively the “e” and “o” keys on a QWERTY keyboard without the numeric keypad or the “y” and “p” keys on a QWERTY keyboard with the numeric keypad). The other half of the participants responded using the opposite stimulus–response mapping. Instruction emphasized both speed and accuracy. No feedback was provided.

There were 24 trials for each combination of the following factors: congruence between gaze direction and target location (congruent vs. incongruent), correspondence between target location and response location (corresponding vs. non-corresponding) and conditions (Mask, No-Mask, Control). A total of 288 trials were presented pseudorandomly (i.e., same random order across participants) across three equal blocks of 96 trials each. A short rest was allowed between blocks. A practice session of 16 trials with only the No-Mask condition was given prior to the beginning of the experimental section.

## Results

Practice trials, errors (3.9% of the total trials) and trials with RTs faster (0.1%) or slower (3.4%) than 2 SD from the individual RT average were not included in the analysis. One participant with RTs average > 3,000 ms was excluded from the sample. The analysis was conducted on 55 participants (40 females, 15 males, 7 left-handed, Mean age = 20.2 years, SD age = 2.5).

The Kolmogorov–Smirnov and Shapiro–Wilk tests indicated that the mean RTs do not follow normal distribution [D(55) = 0.136; *p* = 0.013; W(55) = 0.925, *p* = 0.002]. However, since Skewness and Kurtosis indexes of our dependent variables indicate a slight or moderate departure from normality distribution (see Supplementary Materials for a table with all values), we decided to analyze our data with parametric statistics (see George and Mallery, 2010; Blanca et al., 2017; Field and Wilcox, 2017). A repeated-measures ANOVA was conducted on RTs with *Congruence* (congruent vs. incongruent), *Correspondence* (corresponding vs. non-corresponding), and *Condition* (Mask, No-Mask, Control), as within-subjects factors.

**Figure 3:**
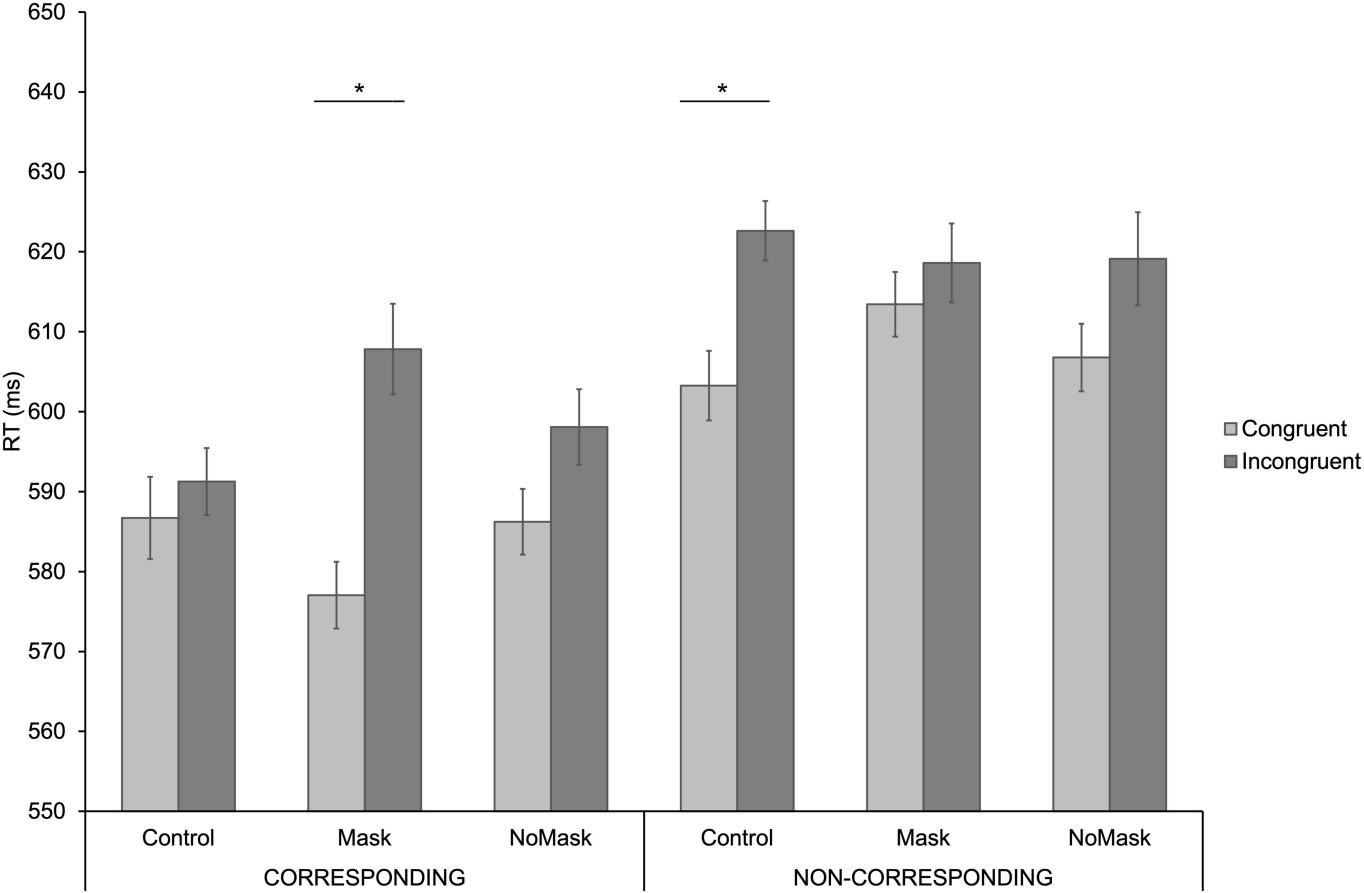
Mean reaction times (RTs) as a function of Condition and Congruence for Corresponding (leftmost panel) and Non-corresponding trials (rightmost panel). Error bars indicate standard errors of the mean adjusted for within-participants designs (Loftus and Masson, 1994). Asterisks denote significant differences.

The analysis revealed a significant main effect of *Congruence* [*F*(1, 54) = 16.364, *MSE* = 32,382,325, *p* < 0.001, η_p_^2^ = 0.233], indicating faster RTs for congruent (M = 595 ms) than incongruent (M = 609 ms) trials, resulting in an overall GCE of 14 ms. There was also a main effect of *Correspondence* [*F*(1, 54) = 35,316, *MSE* = 85,577,699, *p* < 0.001, η_p_^2^ = 0.395], showing faster RTs on corresponding (M = 591 ms) than non-corresponding (M = 613 ms) trials, resulting in an overall Simon effect of 22 ms. The main effect of *Condition* did not reach significance [*F*(1, 54) = 0.652, *MSE* = 637,446, *p* = 0.511, η_p_^2^ = 0.012]. Importantly, there was a significant three-way interaction between *Congruence*, *Correspondence*, and *Condition* [*F*(1, 54) = 5,537, *MSE* = 6,309,092, *p* = 0.007, η_p_^2^ = 0.093], indicating that the GCE differed across conditions and was influenced by correspondence. We found a significant GCE in the Mask condition for Corresponding trials, and in the Control condition for Non-corresponding trials, and non-significant GCE in the No-Mask condition for both Corresponding and Non-Corresponding trials. Specifically, paired sample t-tests showed that for the Mask condition the GCE was 31 ms for the Corresponding trials and 5 ms for the Non-Corresponding trials; for the Control condition the GCE was 5 ms for the Corresponding trials and 19 ms for the Non-Corresponding trials; finally for the No-Mask condition the GCE was 12 ms for the Corresponding trials and 12 ms for the Non-Corresponding trials. See Figure 3 and Table 1.

**Table 1:** Paired simple t-test comparing GCE effect (i.e., Incongruent vs. Congruent trials) in Simon Non-corresponding and Corresponding trials for each experimental condition (i.e., Control, Mask, No-Mask).

Condition				95% confidence interval			
		GCE (ms)	SD	SE	Lower	Upper	*t*	df	*p*	Cohen’s *d*
Control	Simon corresponding	5	47.2	6.4	−8.2	17.3	0.716	54	0.477	0.26
	Simon non-corresponding	19	45.2	6.1	7.1	31.6	3.177	54	0.002	0.05
Mask	Simon corresponding	31	63.5	8.6	13.6	47.9	3.592	54	0.001	0.04
	Simon non-corresponding	5	52.2	7.0	−9.0	19.3	0.734	54	0.466	0.19
No-Mask	Simon corresponding	12	48.9	6.6	−1.4	25.1	1.797	54	0.078	0.11
	Simon non-corresponding	12	46.2	6.2	−0.1	24.8	1.982	54	0.053	0.10

Moreover, we explored whether stimulus–response location (i.e., Simon effect) is influenced by the GCE across conditions. In line with our expectations, we found a strong Simon effect in all experimental conditions regardless of congruence, except for non-significant Simon effect in the Mask condition for incongruent trials. See Table 2. No other main effects or interactions were significant (*F_s_* < 1).

**Table 2:** Paired simple t-test comparing Simon effect (i.e., Non-corresponding vs. Corresponding trials) in GCE Incongruent and congruent trials for each experimental condition (i.e., Control, Mask, No-Mask).

Condition				95% confidence interval			
		SE (ms)	SD	SE	Lower	Upper	*t*	df	*p*	Cohen’ *d*
Control	GCE congruent	17	51.2	6.9	2.7	30.4	2.397	54	0.020	0.15
	GCE incongruent	31	45.1	6.1	19.2	43.6	5.161	54	0.000	0.29
Mask	GCE congruent	36	40.5	5.5	25.4	47.3	6.667	54	0.000	0.35
	GCE incongruent	11	44.7	6	−1.3	22.9	1.79	54	0.079	0.09
No-Mask	GCE congruent	21	52.5	7.1	6.3	34.7	2.9	54	0.005	0.18
	GCE incongruent	21	67.1	9	2.9	39.2	2.324	54	0.024	0.18

## Discussion

The spread of the COVID-19 pandemic has changed our ordinary social behavior, influencing our relationships with others. As a consequence, specific social attentional processes might be more sensitive to important stimuli in the environment aimed at preventing infection. Previous research has extensively investigated attentional processing, showing that both spatial information and social factors are automatically encoded and affect the recognition of perceptual stimuli. However, a growing body of evidence has shown that standard attentional processes could be modulated by the current task and by context-relevant information (e.g., D’Ascenzo et al., 2022; for a discussion, see Lebois et al., 2015).

In the present study, we tested the impact of the facemask adopted to prevent the spread of COVID-19 on social attention through a gaze-cueing paradigm, where stimulus–response correspondence was also investigated. The face stimuli were presented to participants in three different conditions: without the surgical mask (No-Mask), with the surgical mask superimposed on the lower portion of the face (Mask), and with a patch that covered the same area occupied by the mask (Control).

Our results showed an overall GCE across conditions, indicating faster responses when the target was presented in a location congruent to the gaze direction, compared to when it was presented in an incongruent location. This result is consistent with the previous literature on GCE (for a review, see Dalmaso et al., 2020), confirming the role of others’ gaze direction as a social cue to orient attention. In addition, an overall Simon effect emerged, thus when the target was in the same location as the response key position participants were faster than when it was in the opposite location.

Interestingly, the GCE was modulated by condition only when the correspondence between target location and response position was taken into account. Specifically, when target location and response position were on the same side, thus no motor conflict emerged (i.e., corresponding trial), a GCE was evident in the Mask condition and approached significance in the No-Mask condition. In contrast, when target and response were in opposite locations and a motor conflict emerged (i.e., non-corresponding trial), a GCE was found in the Control condition and again approached significance in the No-Mask condition.

Therefore, contrary to our initial predictions, the pattern of results was more complex and partially unexpected. However, the goodness of the experimental design adopted in the present study is suggested by the finding that in the No-Mask condition the classical gaze-cueing effect numerically emerged and did not interact with the Simon effect; thus, this result is in the same direction as those reported in previous studies (e.g., Dalmaso et al., 2012, 2014). Importantly, it is worth noting that in the present study the experiment was administered online. Web-based experiments may add noise to the data compared to the classical lab-based studies in which the experimental setting is more controlled (Sauter et al., 2020; see also Scerrati et al., 2021). Nevertheless, the pattern of the data collected online was in line with the classical gaze-cueing effect since it showed faster reaction time for congruent than incongruent conditions, thus suggesting the reliability of the study. Interestingly, our results only partially replicated evidence coming from a recent study by Dalmaso et al. (2021); published after our data collection was ended and a first version of the paper was drafted. Specifically, the authors explored the impact of face masks on social attention using a standard gaze-cueing task and found that the GCE is not altered as a function of mask condition, that is, a reliable GCE emerged either when stimuli were embedded in faces wearing a mask or not (i.e., Mask and No-Mask condition). However, differently from Dalmaso et al.’s study, we introduced a control condition (i.e., a patch covering the same face area obscured by surgical masks) and the stimulus–response correspondence (i.e., Simon effect) has been also analyzed. Therefore, a direct comparison between the two studies is not appropriate and it would be misleading.

The novelty of our results concerns the Mask and Control conditions in which we found a significant interaction between the GCE and the Simon effect but in opposite directions. Specifically, in the Mask condition, the GCE emerged only in corresponding trials, whereas in the Control condition, the GCE emerged only in non-corresponding trials. This pattern was unpredicted and can be explained by considering how the observer perceived the face covered either by a mask or a patch.

In the case of the Mask condition, there is an interaction between attentional (gaze cueing) and visual motor processes (Simon effect). In keeping with our original hypothesis, the person who is wearing a facemask might be perceived as “trustworthy” (though see Biermann et al., 2021; Malik et al., 2021 for a different interpretation) and this can lead the observer to direct his/her attention in the same direction as the face-masked gaze and not be afraid of approaching him/her. This can explain why in corresponding trials, i.e., those that facilitate action, the GCE emerges and is enhanced; instead, in non-corresponding trials, i.e., those that do not facilitate action, the GCE is inhibited or reduced.

In the case of the Control condition, the face obscured by a patch could likely appear bizarre and suspicious; thus, the seen face might be perceived as “untrustworthy” and potentially dangerous. In this condition, the observer can be inclined to move away from such a face, still monitoring it and paying attention to his/her gaze direction. This can explain the interaction between the gaze-cueing and Simon effect, resulting in an enhancement of the GCE in non-corresponding trials when a patch appears on the face. Indeed, there is evidence that perceiving angry or fearful faces leads to a greater GCE effect than neutral or positive stimuli (e.g., Bayless et al., 2011; Kuhn and Tipples, 2011; Lassalle and Itier, 2013; Pecchinenda and Petrucci, 2016; Carraro et al., 2017; Chen et al., 2021), suggesting that threatening stimuli potentiate automatic orienting to eye gaze.

Our data seem also to be in line with the assumption underlying the conflict monitoring theory (Botvinick et al., 2001), according to which a detected conflict determines an aversive signal that leads to avoidance learning, generating a negative value (Botvinick, 2007; see also Botvinick et al., 2001, 2004). This proposal was supported by several behavioral studies that showed how cognitive conflicts appear to be experienced as aversive events (Dreisbach and Fischer, 2012; Schouppe et al., 2012; see Dreisbach and Fischer, 2015 for review). Indeed, the corresponding trials show a GCE in the Mask condition as the absence of a cognitive conflict together with the presence of the mask on the cueing face may have strengthened participants’ approach behavior (thus facilitating joint orienting). In other words, the mask may have made participants well-disposed towards those people they do not perceive as a threat to their health, encouraging them to orient their attention toward their gaze direction. Therefore at the same time, the absence of a cognitive conflict in the corresponding conditions may have enhanced this favorable behavior, which may have been prevented, instead, in the non-corresponding condition where a conflict emerges (avoidance).

In addition, our findings are in line with previous studies showing that the magnitude of the gaze cueing effect can be modulated by motor information, and sometimes even reversed (e.g., Nummenmaa et al., 2009; Ueda and Kitazaki, 2013). Evidence has been reported that individuals use others’ gaze and head direction not only as a social cue to orient their own attention but also for inferring movement paths that can result in avoidance behaviors. For example, Nummenmaa et al. (2009) conducted an eye-tracking experiment in which participants observed a simulated scenario in which a pedestrian walked directly toward them, and were asked to indicate the direction in which they would orient to skirt the oncoming person by pressing a left or right response button. The authors found that responses were faster for gaze-incongruent than for gaze-congruent trials (i.e., reversed gaze-effect). That is, participants shifted their attention away from the perceived gaze direction to prevent a collision, and tended to fixate longer in the direction of their upcoming movement (i.e., the opposite side the oncoming person was looking at). Crucially, this reverse effect was faster in manual than saccadic responses, indicating that the evaluation of others’ goals affects at first stage one’s own actions and then attention direction. Similar results were obtained by Ueda and Kitazaki (2013) in a study in which participants used mouse movement to avoid collision with a virtual walker who rotated his head leftward or rightward. Specifically, their results showed that when the walker’s head changed direction, participants moved the mouse to the opposite side, thus activating an appropriate movement for collision avoidance. Taken together, these studies suggest that orienting social attention is not always automatically triggered by gaze direction or head orientation, rather further socio-cognitive evaluations related to context can lead to avoidance behaviors (e.g., “reverse” gaze cueing). Thus, gaze direction not only allows us to make inferences about others’ behaviors but is also a useful source of information for our own movement planning.

Interestingly, studies have shown that under certain conditions, attention and social perception influence motor processes. For example, Capellini et al. (2016) investigated the influence of social threat on motor responses using an action observation paradigm in which RTs and the computer mouse trajectories were recorded. The authors found that threatening situations, elicited by an outgroup member and by contextual cues, enhance visual monitoring and interfere with motor responses required by the task. Specifically, when participants faced a stereotypical aggressive outgroup member moving toward a weapon, a delay in response to a target stimulus occurred, suggesting that people allocate their visual attention to this agent and freeze their motor reactions because the context can become potentially menacing.

Similarly, in our study we found that the gaze direction contributes to guiding our own movement in terms of motor responses, depending on social-contextual factors. Face stimuli perceived as safe (i.e., wearing the mask) and that does not expose us to potential risk generate a marked GCE when motor conflict is absent (i.e., corresponding trials) and an approaching behavior is potentially favored, while face stimuli perceived as untrustworthy (i.e., covered by a patch) generates a marked GCE when motor conflict is present (i.e., non-corresponding trials) thus favoring a potential aversive behavior. Interestingly, this is supported by the fact that, when the face was not covered at all (i.e., No-Mask condition) thus conveying no clear or strong information about the social and affective valence of the seen person, the GCE was not affected by motor correspondence, indeed it emerged both in the absence and in the presence of motor conflict. More investigation is required to corroborate our results and to deepen the underpinning mechanisms of the interaction between the GCE and the Simon effect that emerged in the present work. A possible limitation in generalizing our results is that they consider a sample composed of mainly female participants, since previous studies have indicated that females present higher gaze cueing effects and higher sensibility to social cues (e.g., Ciardo et al., 2015; see also Mazzuca et al., 2020).

To conclude, this study provides preliminary evidence of an interaction between gaze-cueing effect and stimulus–response correspondence effect, showing a larger GCE in the corresponding condition for face associated with positive valence that can, thus, enhance potential approaching behavior, and in the non-corresponding condition for face associated to negative valence that can, instead, lead to potential avoidance behavior. Further studies could investigate whether other social characteristics of faces, such as emotions, race, or social status, lead to similar effects. In particular, using a similar paradigm manipulating the social characteristics of the cueing faces could be very informative. For example, using cueing faces validated for trust would be useful to corroborate the hypothesis that the level of trustworthiness of a human face may drive the interaction between our social attention and motor behavior.

## Data availability statement

The original contributions presented in the study are included in the article/Supplementary material, further inquiries can be directed to the corresponding author.

## Ethics statement

The studies involving human participants were reviewed and approved by Ethics committee of the University of Bologna. The patients/participants provided their written informed consent to participate in this study. Written informed consent was obtained from the individual(s) for the publication of any identifiable images or data included in this article.

## Author contributions

CV: conceptualization, methodology, software, data collection, data analyses, and writing—original draft preparation. SD’A: conceptualization, methodology, data analyses, and writing—reviewing and editing. ES: conceptualization, methodology, and writing—reviewing and editing. PR: conceptualization, methodology, writing—reviewing and editing, and supervision. RN: conceptualization, methodology, and supervision. LL: conceptualization, methodology, writing—reviewing and editing, and supervision. All authors contributed to the article and approved the submitted version.

## Conflict of interest

The authors declare that the research was conducted in the absence of any commercial or financial relationships that could be construed as a potential conflict of interest.

## Publisher’s note

All claims expressed in this article are solely those of the authors and do not necessarily represent those of their affiliated organizations, or those of the publisher, the editors and the reviewers. Any product that may be evaluated in this article, or claim that may be made by its manufacturer, is not guaranteed or endorsed by the publisher.

## Supplementary material

The Supplementary Material for this article can be found online at: https://www.frontiersin.org/articles/10.3389/fpsyg.2022.923558/full#supplementary-material
