# Supplementary material for: Wearing the face mask affects our social attention over space
Source: Front Psychol. 2022 Aug 4;13:923558. doi: 10.3389/fpsyg.2022.923558 (PMC9386249; doi:10.3389/fpsyg.2022.923558)
Supplement: Supplementary file 1 [file Data_Sheet_1.docx]

**Table 1.** Paired simple t-test comparing GCE effect (i.e., Incongruent vs. Congruent trials) in Simon Non corresponding and Corresponding trials for each experimental condition (i.e., Control, Mask, NoMask)

|  |  |  | |  |  | **95 % Confidence Interval** | | |  |  | |  |  | |
| --- | --- | --- | --- | --- | --- | --- | --- | --- | --- | --- | --- | --- | --- | --- |
| **Condition** | | | **GCE(ms)** | **SD** | **SE** | **Lower** | | **Upper** | **t** | **df** | | ***p*** | **Cohen’ *d*** | |
| Control | Simon Corresponding | | 5 | 47,2 | 6,4 | -8,2 | 17,3 | | 0,716 | 54 | 0,477 | | 0.26 |  |
|  | Simon Non corresponding | | 19 | 45,2 | 6,1 | 7,1 | 31,6 | | 3,177 | 54 | 0,002 | | 0.05 |  |
| Mask | Simon Corresponding | | 31 | 63,5 | 8,6 | 13,6 | 47,9 | | 3,592 | 54 | 0,001 | | 0.04 |  |
|  | Simon Non corresponding | | 5 | 52,2 | 7,0 | -9,0 | 19,3 | | 0,734 | 54 | 0,466 | | 0.19 |  |
| NoMask | Simon Corresponding | | 12 | 48,9 | 6,6 | -1,4 | 25,1 | | 1,797 | 54 | 0,078 | | 0.11 |  |
|  | Simon Non corresponding | | 12 | 46,2 | 6,2 | -0,1 | 24,8 | | 1,982 | 54 | 0,053 | | 0.10 |  |

**Table 2.** Paired simple t-test comparing Simon effect (i.e., Non corresponding vs. Corresponding trials) in GCE Incongruent and Congruent trials for each experimental condition (i.e., Control, Mask, NoMask).

|  |  |  |  |  | **95 % Confidence Interval** | |  |  | |  |
| --- | --- | --- | --- | --- | --- | --- | --- | --- | --- | --- |
| **Condition** | | **SE(ms)** | **SD** | **SE** | **Lower** | **Upper** | **t** | **df** | ***p*** | **Cohen’ *d*** |
| Control | GCE congruent | 17 | 51,2 | 6,9 | 2,7 | 30,4 | 2,397 | 54 | 0,020 | 0.15 |
|  | GCE incongruent | 31 | 45,1 | 6,1 | 19,2 | 43,6 | 5,161 | 54 | 0,000 | 0.29 |
| Mask | GCE congruent | 36 | 40,5 | 5,5 | 25,4 | 47,3 | 6,667 | 54 | 0,000 | 0.35 |
|  | GCE incongruent | 11 | 44,7 | 6 | -1,3 | 22,9 | 1,79 | 54 | 0,079 | 0.09 |
| NoMask | GCE congruent | 21 | 52,5 | 7,1 | 6,3 | 34,7 | 2,9 | 54 | 0,005 | 0.18 |
|  | GCE incongruent | 21 | 67,1 | 9 | 2,9 | 39,2 | 2,324 | 54 | 0,024 | 0.18 |
