# Supplementary material for: Wearing the face mask affects our social attention over space
Source: Front Psychol. 2022 Aug 4;13:923558. doi: 10.3389/fpsyg.2022.923558 (PMC9386249; doi:10.3389/fpsyg.2022.923558)
Supplement: Supplementary file 2 [file Data_Sheet_2.docx]

| **Variable** |  |  |  | **Statistic** | **SE** |
| --- | --- | --- | --- | --- | --- |
| Control | GCE congruent- Simon Corresponding | *Mean* |  | 586,7052 | 15,11914 |
|  |  | *95% Confidence Interval for Mean* | *Lower Bound* | 556,3931 |  |
|  |  |  | *Upper Bound* | 617,0172 |  |
|  |  | *5% Trimmed Mean* |  | 581,3243 |  |
|  |  | *Median* |  | 559,6043 |  |
|  |  | *Variance* |  | 12572,361 |  |
|  |  | *Std. Deviation* |  | 112,12654 |  |
|  |  | *Minimum* |  | 369,57 |  |
|  |  | *Maximum* |  | 968,94 |  |
|  |  | *Range* |  | 599,36 |  |
|  |  | *Interquartile Range* |  | 132,4 |  |
|  |  | *Skewness* |  | 0,948 | 0,322 |
|  |  | *Kurtosis* |  | 1,326 | 0,634 |
| Mask | GCE congruent- Simon Corresponding | *Mean* |  | 577,0569 | 13,7506 |
|  |  | *95% Confidence Interval for Mean* | *Lower Bound* | 549,4886 |  |
|  |  |  | *Upper Bound* | 604,6252 |  |
|  |  | *5% Trimmed Mean* |  | 571,2019 |  |
|  |  | *Median* |  | 556,9363 |  |
|  |  | *Variance* |  | 10399,352 |  |
|  |  | *Std. Deviation* |  | 101,97721 |  |
|  |  | *Minimum* |  | 369,33 |  |
|  |  | *Maximum* |  | 916,96 |  |
|  |  | *Range* |  | 547,63 |  |
|  |  | *Interquartile Range* |  | 142,42 |  |
|  |  | *Skewness* |  | 1,005 | 0,322 |
|  |  | *Kurtosis* |  | 1,725 | 0,634 |
| No Mask | GCE congruent- Simon Corresponding | *Mean* |  | 586,2321 | 15,17275 |
|  |  | *95% Confidence Interval for Mean* | *Lower Bound* | 555,8126 |  |
|  |  |  | *Upper Bound* | 616,6516 |  |
|  |  | *5% Trimmed Mean* |  | 578,9892 |  |
|  |  | *Median* |  | 552,4021 |  |
|  |  | *Variance* |  | 12661,674 |  |
|  |  | *Std. Deviation* |  | 112,52411 |  |
|  |  | *Minimum* |  | 403,27 |  |
|  |  | *Maximum* |  | 995,44 |  |
|  |  | *Range* |  | 592,17 |  |
|  |  | *Interquartile Range* |  | 136,23 |  |
|  |  | *Skewness* |  | 1,221 | 0,322 |
|  |  | *Kurtosis* |  | 2,1 | 0,634 |
| Control | GCE congruent- Simon Non Corresponding | *Mean* |  | 603,2576 | 14,00266 |
|  |  | *95% Confidence Interval for Mean* | *Lower Bound* | 575,1839 |  |
|  |  |  | *Upper Bound* | 631,3312 |  |
|  |  | *5% Trimmed Mean* |  | 596,887 |  |
|  |  | *Median* |  | 574,7083 |  |
|  |  | *Variance* |  | 10784,092 |  |
|  |  | *Std. Deviation* |  | 103,84648 |  |
|  |  | *Minimum* |  | 396,94 |  |
|  |  | *Maximum* |  | 954,18 |  |
|  |  | *Range* |  | 557,24 |  |
|  |  | *Interquartile Range* |  | 121,16 |  |
|  |  | *Skewness* |  | 1,152 | 0,322 |
|  |  | *Kurtosis* |  | 1,967 | 0,634 |
| Mask | GCE congruent- Simon Non Corresponding | *Mean* |  | 613,4352 | 13,84118 |
|  |  | *95% Confidence Interval for Mean* | *Lower Bound* | 585,6853 |  |
|  |  |  | *Upper Bound* | 641,1851 |  |
|  |  | *5% Trimmed Mean* |  | 608,5761 |  |
|  |  | *Median* |  | 593,8404 |  |
|  |  | *Variance* |  | 10536,803 |  |
|  |  | *Std. Deviation* |  | 102,64893 |  |
|  |  | *Minimum* |  | 405,98 |  |
|  |  | *Maximum* |  | 1014,38 |  |
|  |  | *Range* |  | 608,4 |  |
|  |  | *Interquartile Range* |  | 124,49 |  |
|  |  | *Skewness* |  | 1,174 | 0,322 |
|  |  | *Kurtosis* |  | 3,25 | 0,634 |
| No Mask | GCE congruent- Simon Non Corresponding | *Mean* |  | 606,7744 | 14,63553 |
|  |  | *95% Confidence Interval for Mean* | *Lower Bound* | 577,4319 |  |
|  |  |  | *Upper Bound* | 636,1169 |  |
|  |  | *5% Trimmed Mean* |  | 598,9671 |  |
|  |  | *Median* |  | 587,0165 |  |
|  |  | *Variance* |  | 11780,926 |  |
|  |  | *Std. Deviation* |  | 108,53997 |  |
|  |  | *Minimum* |  | 386,89 |  |
|  |  | *Maximum* |  | 978,53 |  |
|  |  | *Range* |  | 591,64 |  |
|  |  | *Interquartile Range* |  | 130 |  |
|  |  | *Skewness* |  | 1,291 | 0,322 |
|  |  | *Kurtosis* |  | 2,765 | 0,634 |
| Control | GCE incongruent- Simon Corresponding | *Mean* |  | 591,2594 | 15,64635 |
|  |  | *95% Confidence Interval for Mean* | *Lower Bound* | 559,8903 |  |
|  |  |  | *Upper Bound* | 622,6284 |  |
|  |  | *5% Trimmed Mean* |  | 583,4651 |  |
|  |  | *Median* |  | 572,125 |  |
|  |  | *Variance* |  | 13464,454 |  |
|  |  | *Std. Deviation* |  | 116,03643 |  |
|  |  | *Minimum* |  | 384,68 |  |
|  |  | *Maximum* |  | 1007,32 |  |
|  |  | *Range* |  | 622,64 |  |
|  |  | *Interquartile Range* |  | 121,75 |  |
|  |  | *Skewness* |  | 1,246 | 0,322 |
|  |  | *Kurtosis* |  | 2,449 | 0,634 |
| Mask | GCE incongruent- Simon Corresponding | *Mean* |  | 607,8195 | 17,6084 |
|  |  | *95% Confidence Interval for Mean* | *Lower Bound* | 572,5168 |  |
|  |  |  | *Upper Bound* | 643,1222 |  |
|  |  | *5% Trimmed Mean* |  | 597,3847 |  |
|  |  | *Median* |  | 566,2092 |  |
|  |  | *Variance* |  | 17053,057 |  |
|  |  | *Std. Deviation* |  | 130,58735 |  |
|  |  | *Minimum* |  | 402,08 |  |
|  |  | *Maximum* |  | 1168,27 |  |
|  |  | *Range* |  | 766,18 |  |
|  |  | *Interquartile Range* |  | 155,29 |  |
|  |  | *Skewness* |  | 1,754 | 0,322 |
|  |  | *Kurtosis* |  | 5,371 | 0,634 |
| No Mask | GCE incongruent- Simon Corresponding | *Mean* |  | 598,0939 | 14,57701 |
|  |  | *95% Confidence Interval for Mean* | *Lower Bound* | 568,8688 |  |
|  |  |  | *Upper Bound* | 627,3191 |  |
|  |  | *5% Trimmed Mean* |  | 592,1732 |  |
|  |  | *Median* |  | 579,913 |  |
|  |  | *Variance* |  | 11686,911 |  |
|  |  | *Std. Deviation* |  | 108,10602 |  |
|  |  | *Minimum* |  | 413,75 |  |
|  |  | *Maximum* |  | 994,72 |  |
|  |  | *Range* |  | 580,98 |  |
|  |  | *Interquartile Range* |  | 115,41 |  |
|  |  | *Skewness* |  | 1,136 | 0,322 |
|  |  | *Kurtosis* |  | 2,198 | 0,634 |
| Control | GCE incongruent-Simon NonCorresponding | *Mean* |  | 622,6286 | 13,72485 |
|  |  | *95% Confidence Interval for Mean* | *Lower Bound* | 595,1119 |  |
|  |  |  | *Upper Bound* | 650,1452 |  |
|  |  | *5% Trimmed Mean* |  | 617,7154 |  |
|  |  | *Median* |  | 594,0652 |  |
|  |  | *Variance* |  | 10360,428 |  |
|  |  | *Std. Deviation* |  | 101,78619 |  |
|  |  | *Minimum* |  | 431,16 |  |
|  |  | *Maximum* |  | 918,7 |  |
|  |  | *Range* |  | 487,54 |  |
|  |  | *Interquartile Range* |  | 133,44 |  |
|  |  | *Skewness* |  | 0,809 | 0,322 |
|  |  | *Kurtosis* |  | 0,833 | 0,634 |
| Mask | GCE incongruent-Simon Non Corresponding | *Mean* |  | 618,6031 | 15,33477 |
|  |  | *95% Confidence Interval for Mean* | *Lower Bound* | 587,8587 |  |
|  |  |  | *Upper Bound* | 649,3475 |  |
|  |  | *5% Trimmed Mean* |  | 611,2475 |  |
|  |  | *Median* |  | 585,9087 |  |
|  |  | *Variance* |  | 12933,541 |  |
|  |  | *Std. Deviation* |  | 113,72573 |  |
|  |  | *Minimum* |  | 429,47 |  |
|  |  | *Maximum* |  | 1037,51 |  |
|  |  | *Range* |  | 608,04 |  |
|  |  | *Interquartile Range* |  | 130,97 |  |
|  |  | *Skewness* |  | 1,316 | 0,322 |
|  |  | *Kurtosis* |  | 2,605 | 0,634 |
| No Mask | GCE incongruent-Simon Non Corresponding | *Mean* |  | 619,1119 | 16,39038 |
|  |  | *95% Confidence Interval for Mean* | *Lower Bound* | 586,2512 |  |
|  |  |  | *Upper Bound* | 651,9726 |  |
|  |  | *5% Trimmed Mean* |  | 607,7576 |  |
|  |  | *Median* |  | 594,2215 |  |
|  |  | *Variance* |  | 14775,452 |  |
|  |  | *Std. Deviation* |  | 121,55432 |  |
|  |  | *Minimum* |  | 404,66 |  |
|  |  | *Maximum* |  | 1135,66 |  |
|  |  | *Range* |  | 731 |  |
|  |  | *Interquartile Range* |  | 125,94 |  |
|  |  | *Skewness* |  | 2,012 | 0,322 |
|  |  | *Kurtosis* |  | 6,452 | 0,634 |
